# Multiple Object Tracking Without Pre-attentive Indexing

**DOI:** 10.1162/opmi_a_00128

**Published:** 2024-03-26

**Authors:** Shubhamkar Ayare, Nisheeth Srivastava

**Affiliations:** Department of Cognitive Science, IIT Kanpur, Kalyanpur, Kanpur

**Keywords:** multiple object tracking, visual indexing theory, computational modeling

## Abstract

Multiple object tracking (MOT) involves simultaneous tracking of a certain number of target objects amongst a larger set of objects as they all move unpredictably over time. The prevalent explanation for successful target tracking by humans in MOT involving visually identical objects is based on the Visual Indexing Theory. This assumes that each target is indexed by a pointer using a non-conceptual mechanism to maintain an object’s identity even as its properties change over time. Thus, successful tracking requires successful indexing and the absence of identification errors. Identity maintenance and successful tracking are measured in terms of identification (ID) and tracking accuracy respectively, with higher accuracy indicating better identity maintenance or better tracking. Existing evidence suggests that humans have high tracking accuracy despite poor identification accuracy, suggesting that it might be possible to perform MOT without indexing. Our work adds to existing evidence for this position through two experiments, and presents a computational model of multiple object tracking that does not require indexes. Our empirical results show that identification accuracy is aligned with tracking accuracy in humans for tracking up to three, but is lower when tracking more objects. Our computational model of MOT without indexing accounts for several empirical tracking accuracy patterns shown in earlier studies, reproduces the dissociation between tracking and identification accuracy produced earlier in the literature as well as in our experiments, and makes several novel predictions.

## INTRODUCTION

**Figure 1.**
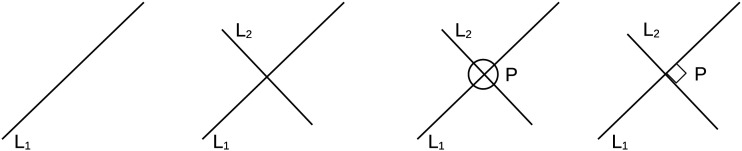
Illustration of how the process of noticing things in the environment happens in a step-by-step manner using a geometric reasoning task. (i) Notice the line *L*_1_. (ii) Notice another line *L*_2_. (iii) Notice that the *L*_1_ and *L*_2_ intersect at *P*. However, notice that this also requires noticing that the *L*_1_ and *L*_2_ are the same ones that we had noticed when we were processing step (i) and (ii). (iv) Notice that the angle at *P* is actually a right angle. Again, notice that this also requires noticing that this *P* is the same *P* that we had considered in (iii).

Object tracking is essential for any system or organism that lives through time. An example cited by Pylyshyn (2007, 2009) concerns the construction of a system that Pylyshyn et al. (1978) attempted to build in order to perform geometrical reasoning in a psychologically plausible manner (Figure 1). Like a human, such a system was constrained to notice various aspects of the image over time without having access to all the aspects simultaneously. In particular, this required positing that there is a mechanism by which one can say that two visual elements at two different points of time refer to the same “thing”. Two examples based on Figure 1 (iv) include: (a) noticing that the intersection that has a right angle in (iv) is the same intersection formed by the two straight lines *L*_1_ and *L*_2_ which were noticed earlier in (iii), or also, (b) the two straight lines are indeed the same ones that were drawn and noticed yet earlier in steps (i) and (ii). Eventually, this, amongst other things, led Pylyshyn (1989) and Pylyshyn and Storm (1988) to consider the existence of a non-conceptual mechanism that can solve the correspondence problem of identifying which relevant visual element at a time point *t* is which relevant visual element at another time point *t* + Δ*t*.

This mechanism is Pylyshyn’s famous Fingers of INSTantiations (Pylyshyn, 1989, 2001) proposal, which suggests the existence of more-than-one pointers^1^ providing parallel non-conceptual access to visual elements through time. A FINST has been described as a sticky reference to visual elements that keeps pointing to the elements even as their location or other properties change over time. The FINST uses only the non-encoded (non-represented, non-conceptual) properties to maintain the reference, not the encoded (represented) properties.

**Figure 2.**
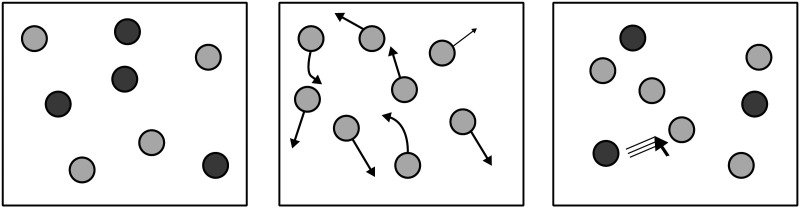
A Multiple Object Tracking (MOT) task can be identified by three phases: Left - target designation phase, in which the targets are highlighted either by using a different color or blinking or both. Center - tracking phase, in which the targets are visually distinct from the distractors, and all the object move around potentially randomly on the display. Right - response phase, in which the participant is asked to select all the objects that they think are the targets.

MOT tasks in the literature can be broadly classified into two kinds: (i) those involving visually identical objects (ii) those involving visually distinct objects. In MOT tasks with visually distinct objects, both indexing as well as the visual distinctiveness of the target objects can be used to perform tracking, e.g., Horowitz et al. (2007) suggest a significantly greater capacity to track visually distinct objects than visually identical objects. Since our work focuses on the nature of indexing, our work uses MOT tasks involving visually identical objects. Thus, we focus on a classic variant of MOT (Figure 2) that involves the tracking of *n* target objects amongst *m* visually identical distractor objects, all moving randomly.

Following Vul et al. (2009), we adopt a definition of tracking accuracy as the percentage of the tracked objects that are targets at the end of the trial. Similarly, success in identification is measured using identification (ID) accuracy, defined as the percentage of tracked objects that are labelled correctly. For both measures, the number of targets is the same as the number of tracked objects in the experiments we consider. In particular, for an MOT trial with *n* targets and *m* distractors, suppose that the participant identifies *k* ≤ *n* targets correctly, and the remaining (*n* − *k*) objects that the participant identifies as targets are actually distractors. Then, the tracking accuracy^2^ is given by *k*/*n*. Each target may be given a label to check its identity maintenance by the participant. The labels are visible only at the start of the trial.

According to the FINST-based explanation of MOT (Pylyshyn, 2004), at the start of the trial, the participant forms an association between each target (thus, its identity) and its label. During the trial, the labels disappear and the objects move around. The participant maintains each tracked object as an individual entity, and does not confuse its individuality with other tracked objects. Then, at the end of the trial, the participant retrieves from memory the label associated with each tracked object, and responds accordingly. Suppose that the participant labels *p* ≤ *n* targets correctly. Then, the ID accuracy is given by *p*/*n*.

Contemporary computational models of MOT of visually identical objects are also usually premised on the existence of FINST-like non-conceptual pre-attentive indexes in the visual system (Alvarez & Franconeri, 2007; Oksama & Hyönä, 2008; Srivastava & Vul, 2016)^3^. These indexes enable the models to establish a correspondence between the representation of an object that is being noticed at the current moment and an earlier one. Pylyshyn’s work indicates that the number of FINSTs available in human visual perception to be between 4–5. However, Alvarez and Franconeri (2007) have shown that, MOT task participants are able to track not just 4 but even 8 objects with 94% accuracy (at sufficiently low movement speeds). Their experiments also noted that the speed-threshold for 94% accuracy deteriorated smoothly with increasing number of targets happens smoothly rather than abruptly. Overall, these results suggest either that the number of FINSTs is flexible or that MOT might be possible without FINSTs.

The strongest prior evidence against the involvement of FINSTs in MOT comes from the experiments in Pylyshyn (2004), whose results suggest a disparity between ID and tracking accuracy. In this case, the ID accuracy is worse than, as well as deteriorates more rapidly than tracking accuracy as a function of tracking duration. For instance, for a tracking duration of 10 seconds, tracking accuracy is 70% while the ID accuracy is 30%. Since FINSTs are meant to provide an apriori incorruptible mechanism to establish a correspondence between two visual elements, one being accessed currently while the other that was present at an earlier point of time, they do not offer a natural explanation for the confusion in object identities seen in Pylyshyn (2004).

In the rest of the paper, we firstly discuss what tracking with and without indexes means to set the stage for our work. There, we also outline a model of tracking that does not use indexes. We also elaborate on this model computationally after discussing the two experiments, and note that the model can account for several specific empirical results documented empirically, viz.

1. decrease in tracking accuracy with increasing number of targets
2. decrease in object speed thresholds corresponding to a particular accuracy with increasing number of targets
3. dependence of tracking accuracy on crowding aka object speed as measured in terms of scene-widths per second rather than absolute speeds
4. variation in tracking and ID accuracy disparity with increasing trial duration
5. variation in tracking and ID accuracy disparity with increasing number of targets

While accounting for these, our modeling work^4^ suggests some model-limitations, several interesting predictions about tracking, as well as on some constraints involved in attending to multiple objects simultaneously^5^.

## TRACKING WITH AND WITHOUT INDEXES

Before presenting our model, we discuss what it means to say that indexes are involved in tracking and, thus, what an index-less model should look like.

Both index-based and index-less accounts involve the processing and use of the instantaneous location information of the objects by the visual system for the MOT task. The critical difference between the two accounts is at what stage of information processing the location information gets used for tracking. The indexes in the Visual Indexing Theory are a part of the early visual system (Pylyshyn, 2001). They use the location information soon after the early visual system processes it (Pylyshyn, 2007) (chapter 3). This process is stimulus-driven and automatic. No representations or concepts are involved^6^—the location information is necessarily non-conceptual^7^. On the other hand, an index-less account of tracking requires the location information to be represented and made available to the cognitive system before it gets used for tracking.

**Figure 3.**
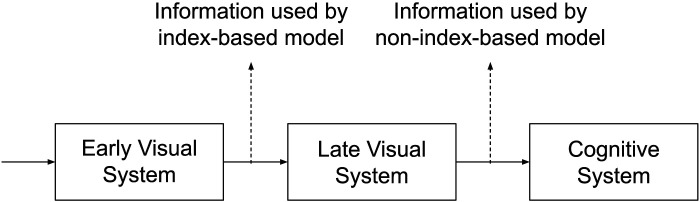
The difference in information processing of an index-based account of MOT vs. a non-index-based account of MOT.

Overall, the early visual system processes the location information from the retinal stimulus into non-conceptual location information. An indexing-based account uses this location information for tracking. The visual system further processes and encodes this information, making it available to the cognitive system. An index-free account uses this location information. This process is also summarized in Figure 3.

While presenting support for FINSTs, Pylyshyn and Storm (1988) had reported a computational model of tracking with assumptions based on the empirical literature available then. This model—the Serial Tracking Algorithm—was based on the updation of encoded (represented) location information by the cognitive system and was, therefore, an index-less model. However, the tracking accuracy possible with this Serial Tracking Algorithm was about 30%. On the other hand, the human participants had a much better tracking accuracy of 87%. This disparity led them to accept that tracking happens through a pre-attentive parallel mechanism that does not require location information to be encoded—the process of using the encoded location information is too inefficient. This pre-attentive mechanism is precisely that of the FINSTs or the Visual Indexing Theory.

Pylyshyn and Storm (1988) report that the index-less Serial Tracking Algorithm could not account for the human tracking accuracy even after several augmentations. However, we propose that these assumptions are inconsistent with some of the evidence collected since then. Firstly, the Serial Tracking Algorithm assumes that focal attention moves continuously from one location to another, passing through all locations between objects. However, Egeth and Yantis (1997) have indicated that the movement of attention is quantal. Secondly, the algorithm does not acknowledge events happening at rates beyond 10 Hz (such as the phi phenomena, Wertheimer, 1912) because even when velocity information was used for tracking, the dwell time was assumed to be about 100 ms.

Moreover, Scholl et al. (2001) report that in an MOT task, participants indeed have cognitive access to the location information of the targets. In their experiment, all the objects disappeared briefly at the end of a MOT trial. After 200 ms, all but one object became visible again. The participants had to indicate the location of the disappeared object and whether it was a target or a distractor. They noted that participants’ accuracy was significantly better for targets than distractors. Suppose the location information was cognitively inaccessible. In that case, equal disappearance-detection accuracy would be expected for both targets and distractors. Contrary evidence, thus, indicates that the location information is cognitively accessible. Cognitive access requires that the location information is accessed as representations. These experiments raise questions about the critical assumption behind index-based explanations of tracking, that the location information is used without it becoming accessible to the cognitive system. Stronger evidence in support of the position that the early visual system can perform MOT—per the Visual Indexing Theory—can be obtained by studying MOT in participants with blindsight; however, we know of no such experiment.

To summarize (see Table 1), if an account of tracking involves updates of representations then that account would qualify as an index-less account of tracking. On the contrary, if no representations are involved, and tracking takes place through non-encoded (non-represented) information using FINST-based pointers, then that will be an index-based account of tracking.

**Table 1.** A comparison of an index-based vs. index-less account of MOT.

	Index-based account	Index-less account
Tracking performed by	Early Visual System	Cognitive System
Location information is	Non-conceptual	Conceptual or non-conceptual
Representations are	Not involved	Involved

### MOTUAF: Multiple Object Tracking as Updates of Attended Features

The crux of the index-less tracking approach we propose is two retinotopic maps, one corresponding to the actual features of the objects themselves and another corresponding to the attended features, which corresponds to the reportable features of the objects. Unlike Blaser et al. (2000), who explored tracking an object through the feature spaces of color, orientation, and spatial frequency, our work limits itself to studying MOT in the location space. In this case, the existence of a map of attended locations as distinct from the map of the actual locations of the objects is also evident through the reports by Howard and Holcombe (2008) and Howard et al. (2011), which suggest a dissociation between the representations of positions of the targets and the actual positions of the targets themselves.

The exact structure of each of the two maps will remain a topic of future work. However, in general, we consider each map to be comprised of a number of grid cells corresponding to the different locations on the retinotopic map. Each grid cell of the first map indicates the presence or absence of an object and only their number but not identity. Each grid cell of the second map indicates the presence or absence of attention and, equivalently, the represented object locations.

The updates to this first retinotopic map of objects are stimulus-driven, parallel, and effortless. Howard and Holcombe (2008) and Howard et al. (2011) indicate that the dissociation, aka the time lag between the position representations of targets and their actual locations, increases with an increasing number of targets. Given that, we expect that the updates to the map of attended locations are effortful and, thus, happen sequentially or in parallel with a constrained resource. The sequential nature of updates is also evident through the findings by Holcombe and Chen (2013) who have noted that the tracking limit for 2 targets is almost one-half that of 1 target, while the tracking limit for 3 targets is almost one-third of 1 target.

**Figure 4.**
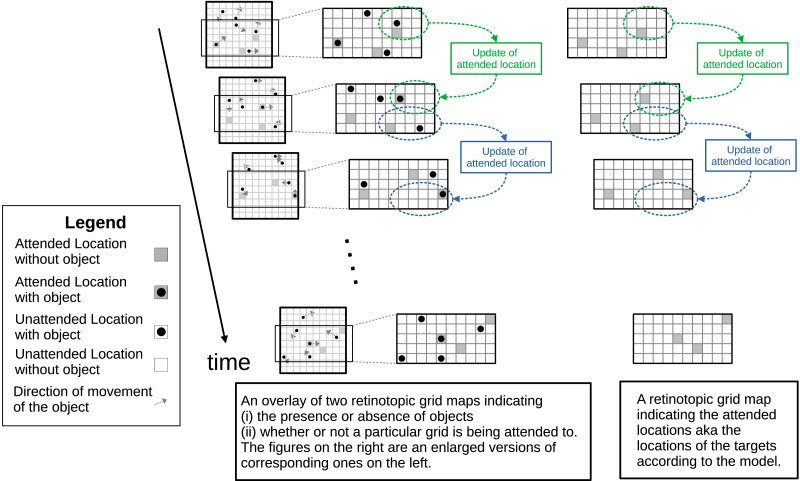
An illustration of our model with the shaded grids in the retinotopic grid map indicating the attended locations aka locations supposed to correspond to targets, along with the presence / absence of objects at each location. While indexes provide a location-independent means to individuate two objects, there exist no such location-independent way to distinguish between two attended locations.

Thus, we propose that the updates of the attended grid cells on the second map constitute the constrained resource. In this framework, multiple object tracking consists of maintenance of attended features (in our case, locations) so that they keep corresponding to objects as far as possible (Figure 4). Such maintenance involves an update of attended features (locations) that no longer correspond to any objects to other nearby features (locations) that correspond to an object. The greater the number of targets, the less frequent the updates to each target’s relevant feature; thus, the maintenance is worse. This assumption is a specialization of more generic resource constraints found in the works of Alvarez and Franconeri (2007) and Srivastava and Vul (2016). Alvarez and Franconeri (2007) demonstrate that tracking is limited not by a fixed number of indexes as the FINST theory suggests but by a shared resource. They relate this shared resource to the precision with which an object is selected; thus, more the allocated resource, more is the precision with which the object is selected and lesser the tracking errors. Srivastava and Vul (2016) incorporate this explanation in a computational model, which they show can explain trial level variations in MOT performance across different participants. In MOTUAF, the spatial precision arises naturally from the updates of attended features; the update process is elaborated under “MOTUAF Attention updates” under the section on Computational Modeling.

Most importantly, unlike FINST (Pylyshyn, 1989, 2009) and MOT models based on FINSTs, all attended locations in our model are indistinguishable except by their locations. The contrast is that FINST-based models enable individuating objects without considering their visual features or locations because a different FINST indexes each object.

We assume that the total number of attended locations can vary within an individual based on task requirements. We expect an upper limit to the number of attended locations; it is certainly above 4 given the evidence that subjects can track as many as 8 objects with 94% accuracy, moving at low velocities (Alvarez & Franconeri, 2007). We speculate that the limit should be related to one’s working memory capacity, a question future work should address.

### Identity Maintenance Without Indexes

MOT, as proposed here, does not allow the system to access object locations at two different time steps and, thus, does not require the system to solve the correspondence problem.

To keep track of IDs, we therefore propose that there also exists a separate sequence of IDs. One possible strategy for maintaining such a sequence is to keep reciting the sequence. To match objects to IDs, one needs to go over the attended objects in some spatial sequence while reciting their IDs in that exact sequence.

Alongside the sequence of IDs, there also exists another sequence corresponding to the attended locations. Such a sequence can be obtained by sorting the attended locations in a non-decreasing order of x-and-y coordinates. The ID sequence consists of IDs of the objects arranged according to these attended locations. ID maintenance, then, involves maintaining the explicit correspondence between the sequence of IDs and the sequence of attended locations. ID errors arise when one fails to update this correspondence.

Our proposed mechanism for object identification yields a concrete testable prediction: as the number of targets increases, the length of the ID sequence and the attended location sequence increases. Thus, updating them to maintain the correspondence becomes harder and thus the tracking-ID accuracy disparity should increase with increasing number of targets. Experiment 1, discussed in the next section, tests precisely this prediction.

## EXPERIMENT 1

Pylyshyn (2004) have previously shown an ID-tracking accuracy disparity with respect to tracking duration for MOT tasks involving visually identical objects. However, no earlier work has examined how this disparity varies with changing number of targets. Based on earlier results, (Pylyshyn & Storm, 1988), we expect the tracking (as well as the ID) accuracy to decrease with the increasing number of targets. However, in addition to these two main effects, our proposed explicit ID maintenance mechanism predicts that the participants will not be able to do correspondence updates as rapidly as required by the changing spatial sequence of targets. Therefore, the ID accuracy will degrade more rapidly than tracking accuracy with the increasing number of targets. We decided to test this prediction against data from human participants. This MOT experiment involves both ID and tracking tasks, randomly varying the number of targets across the trials.

### Participants

13 participants (8 men, 5 women) participated in the experiment. All had normal or corrected to normal vision, and none were colorblind. An IRB approved the protocol for the experiment.

### Procedure

10 practice trials, followed by 80 main trials were employed. The number of targets varied from 1 to 8 across the trials. 10 trials for each case of number of targets from 1 to 8 accounted for the 80 trials. Each trial had 14 objects. The tracking duration in each trial was 5 seconds but the trials were self-paced and randomized. In each trial, the participant had to select all the targets and indicate the ID number for each of the target. This procedure was similar to that employed in Experiment 4 of Pylyshyn (2004).

### Materials

For the purposes of the experiment, the visually identical objects comprised of small circles with a diameter of 10 pixels. The participants sat at a distance of about 60 cm from the display. Thus, each object subtended an angle of 0.23° at the retina, and the objects were allowed to move in a 720 × 720 pixels square area whose diagonal subtended an angle of 23.5° at the retina.

### Results

**Figure 5.**
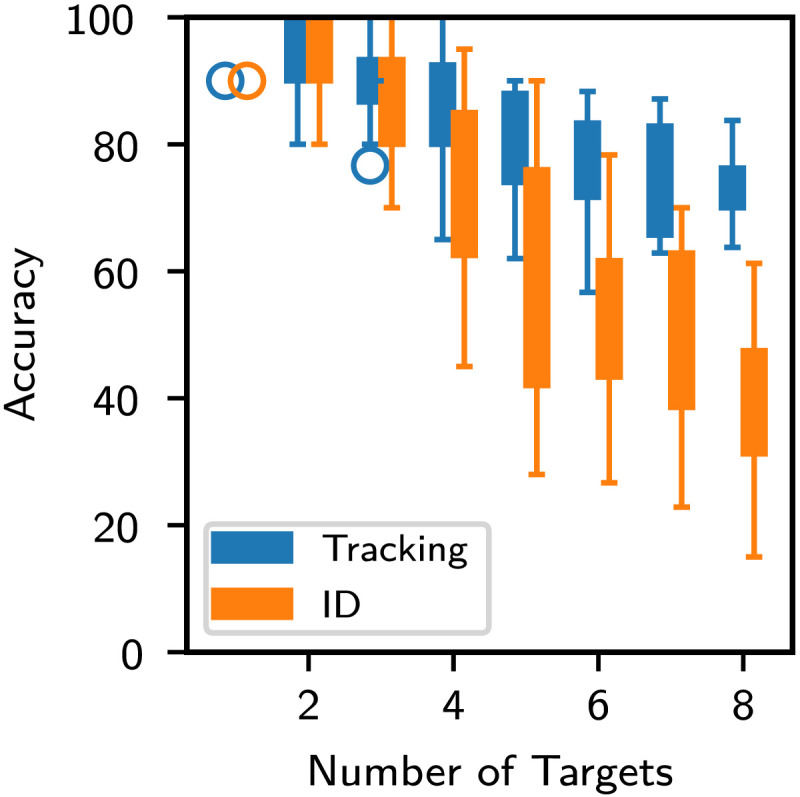
Tracking and ID accuracy of participants with increasing number of targets in Experiment 1.

Repeated measures two-way ANOVA was conducted with tracking-vs-ID accuracy as one factor, and the number of targets as the second factor. The results were as per the expectations discussed above (Figure 5)—there was a significant interaction effect [*F*(3.46, 41.47) = 37.638, *p* = 1.39 × 10^−12^] as well as significant main effects for number of targets [*F*(7, 84) = 70.845, *p* = 1.49 × 10^−32^] and task [*F*(1, 12) = 113.476, *p* = 1.80 × 10^−7^].

**Figure 6.**
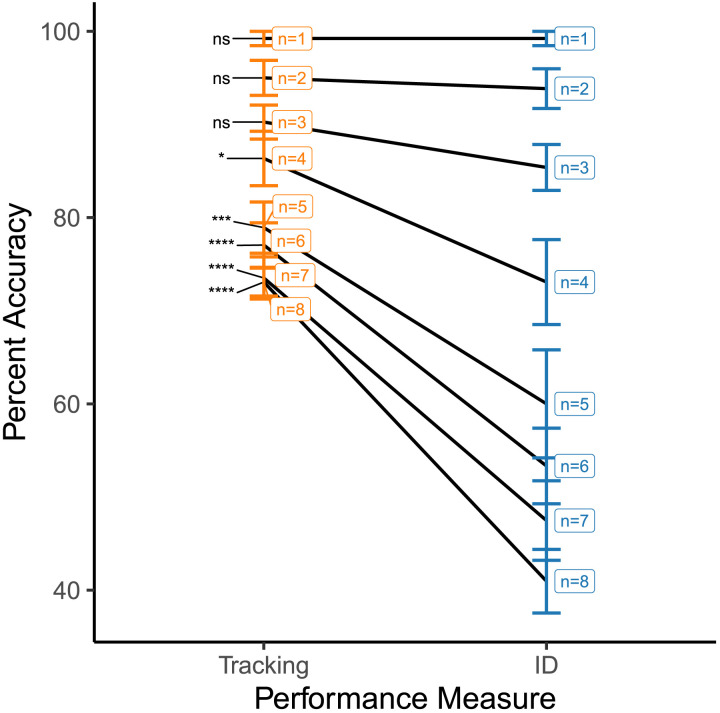
Pairwise comparison of average tracking and ID accuracy across different number of targets. ‘n’ denotes the number of targets. Error bars denote 1 SEM. Comparisons marked ‘ns’ are statistically insignificant, those marked ‘*’ have *p* < 0.05, ‘**’ have *p* < 0.01, ‘***’ have *p* < 0.001, ‘****’ have *p* < 0.0001.

Figure 6 summarizes the tracking-ID accuracy disparity for each of the number of targets. The p-values are adjusted according to Bonferroni correction with a factor of 8. The comparison is also performed in terms of Bayes’ Factor in Table 2 (Morey et al., 2015). When number of targets is 4 or more than 4, the disparity between tracking and ID accuracies is statistically significant at an *α*-level of 0.05 and a Bayes factor threshold of 30.

**Table 2.** Pairwise comparison of average tracking and ID accuracy across different number of targets using Bayes’ Factor. The null hypothesis is that both the accuracies are equal; the alternate hypothesis is that both are different. The Bayes’ Factor expresses the support for the alternate against the null—a high value indicates greater support for the alternate.

Number of Targets	1	2	3	4	5	6	7	8
Bayes’ Factor	NA	0.61	17.7	173	91.6	9.2 × 10^3^	1.5 × 10^5^	3.7 × 10^5^

### Discussion

Experiment 1 checked how the disparity between tracking and ID accuracy varies as the number of targets increase from 1 to 8. Empirical evidence from this experiment indicated that the ID accuracy was indeed worse than tracking accuracy when the number of targets 4 or more. Further, we note that in Pylyshyn (2004), as well as our experiment 1, objects were allowed to come arbitrarily close to each other and cross paths. Trick and Pylyshyn (1994) have used indexing to explain subitizing. This phenomenon involves the rapid and accurate counting of the number of items when there is a small number of them. Such counting is slow and prone to errors when the number of items exceeds four or five. Trick and Pylyshyn (1994) explain this phenomenon by proposing that when the number of items is 4–5 or less, counting them involves merely counting the number of active indexes assigned pre-attentively and automatically. However, when the number of items exceeds 4–5, then indexes need to be reassigned, and participants need to keep track of which items have been already counted. This process is slow and prone to errors. Intriligator and Cavanagh (2001) have noted that there are certain specific conditions, aka conditions of attentional individuation, in which the objects can capture the FINSTs even when the number of objects is 4 or less. In particular, when the objects are close to each other, participants can resolve them visually but not select them individually using attention. We suspect that these same conditions are the conditions that allow FINSTs to remain bound to their referents.

**Figure 7.**
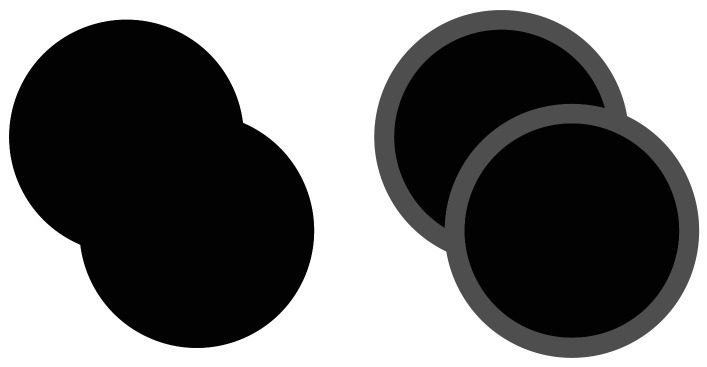
Viswanathan and Mingolla (2002) provide empirical evidence that the absence of T-junction cues (left) corresponding to the overlap of two objects deteriorates tracking accuracy in an MOT task. On the other hand, the presence of such cues (right) does not significantly reduce accuracy.

The experiments in Pylyshyn (2004) allowed objects to overlap, but they also provided T-junction cues (Figure 7) corresponding to the object overlaps. Viswanathan and Mingolla (2002) have indicated that the provision of depth cues such as T-junctions for overlapping objects does not deteriorate tracking accuracy significantly. However, it is unclear if such cues also prevent the disruption of subitizing discussed above (Intriligator & Cavanagh, 2001). Pylyshyn (2004) acknowledges this for the posthoc analysis of experiment 4.

Thus, to find out if it is rather the case that the worse ID accuracy in the cases when number of targets was 2, 3, or 4 was a result of FINST reassignments enabled by the close encounters of objects, we report a second experiment that was specifically designed to keep the objects separate from each other while also maximizing the possibility of ID errors.

## EXPERIMENT 2

The second experiment consisted of two tasks designed to obtain tracking-ID accuracy dissociation without violating the conditions for attentional individuation discussed above. In the first task, we asked participants to choose a speed limit for the objects at which they were barely able to track them. We used these per-participant speed limits in the second task to choose trials specifically designed to confuse identities. These trials were based on novel motion dynamics based on the magician’s shell game. In all the trials, objects always remained at a minimum visual-angle separation of 4 degrees from each other. The motivation for choosing this separation has been elaborated in the Materials section below, while the motion dynamics are elaborated in the discussion of Task 2 under the section on Procedure.

By preventing objects from approaching each other, we intended to eliminate the possibility of tracking errors arising from index reassignments. In this case, an indexing-based account would predict that the participants will not make ID errors in the second task. Contrapositively, ID errors on the second task would raise concerns for the indexing-based account. Explaining ID errors would necessitate positing that indexes are corruptible or require a non-indexing based account of MOT—*some* non-indexing based account, even if not the one we propose in this paper.

### Participants

12 participants (7 men, 5 women) participated in the experiment. All had normal or corrected to normal vision, and none were colorblind. An IRB approved the protocol for the experiment.

### Materials

For the experiment, the visually identical objects were small circles with a diameter of 30 pixels. The participants sat about 40 cm from a full HD display with a diagonal of 21-inch length. Thus, each object subtended an angle of 1° at the retina. The objects were restricted to move in a 1080 × 720 pixels square area, which subtended an angle of 37.4° × 25° at the retina.

Our decision to use the minimum object separation of 4 degrees is based on the second experiment in Intriligator and Cavanagh (2001). Preventing index reassignments requires that the minimum distance between the objects be greater than the attentional resolution. Intriligator and Cavanagh (2001) report a 75% accuracy in the stepping task corresponded to an attentional resolution of (i) 3 arc min at the fovea (ii) 2° at an eccentricity of 15°. Attentional resolution corresponding to an eccentricity of 19° would be ideal for our task; however, in its absence, we use the attentional resolution corresponding to 15° eccentricity.

Note also that we are interested in near-100% accuracy rather than merely 75%. From the Figure 11 of Intriligator and Cavanagh (2001), one may note that halving the density in the stepping task at 15° eccentricity would correspond to an accuracy over 90%. This halving would correspond to an attentional resolution of 4°. A similar separation has also been used in Alvarez and Franconeri (2007) who report a minimum separation of 4° for a display 30° × 24°) and a tracking accuracy of 94%. Participants were asked to fixate on a central cross, but they were told they can move their eyes if it helps them with the task. Thus, we expect the separation of 4° to support attentional resolution and preventing index reassignments.

### Procedure

Both tasks employed trials, each of which had 2, 3, or 4 targets. Each trial of both tasks has a target designation phase, during which the targets were visually distinct (green) from the distractors (red). Following this was the tracking phase, during which all the objects were visually identical (white) and moved about on the display. Once all the objects stopped moving, the response phase began in which the participant had to indicate their response, which depended on the task and has been elaborated below. In all the trials, all pairs of objects were separated by 120 pixels on the display, translating to about 4 degrees of visual angle in the periphery.

#### Task 1: Maximum Speed for Error-Free Tracking.

The first task was a calibration task intended to find the maximum speeds at which the participants could track objects under the circumstances of the experiment. It employed three blocks, each differing from the other in terms of the number of targets. The sequence of blocks was counterbalanced across the participants. This meant that if one participant performed calibration in the order of blocks containing 2 targets, 3 targets, 4 targets, then another participant would perform the calibration in the order containing 4 targets, 2 targets, 3 targets, and so forth for the other permutations of the sequence [2, 3, 4].

In this task, there were 8 objects, and 2, 3, or 4 targets. The object motion dynamics followed the Ornstein-Uhlenbeck dynamics with the restriction that any two objects will always be at least 120 pixels aka 4° away from each other. The participants were told that in this task, they had to identify the maximum speed at which they can perfectly track all the targets. They were asked to use the up and down arrow keys of the keyboard to adjust the objects’ speeds.

Pressing the ‘up’ arrow key once increased *σ* for the ongoing trial by 0.5 up to a maximum of *σ*_max_ = 6.0, and ‘down’ arrow key decreased *σ* by 0.5 down to a minimum value of *σ*_min_ = 0.5. At the start of the trial, *σ* was initialized to *σ*_0_ = 1.0.

If the participant was successful in identifying all the targets in the current trial *T* with *σ* = *σ*^*T*^, *σ*^*T*+1^ for the subsequent trial *T* + 1 was randomly set to either the same *σ*^*T*^ as the current trial, or *σ*^*T*^ + 0.5, or *σ*^*T*^ − 0.5, all three with equal probability of 1/3 each, upper bounded by *σ*_max_ = 6.0 and lower bounded by *σ*_min_ = 0.5. On the other hand, if a participant was unsuccessful in identifying all the targets, *σ*^*T*+1^ was reset to *σ*_0_ = 1.0.

For each block, calibration was said to be successful if the participant correctly identified all the targets for 5 consecutive trials, and the *σ* value across these 5 trials varied by a maximum of 0.5. Once this condition was met, the participant was informed that calibration for that particular target number was successful, and the next block was initiated. Once all the blocks were completed, the experiment moved on to the next task.

#### Task 2: ID and Tracking Error Dissociation.

Task 1 ensured that for each case involving the number of targets as 2, 3, or 4, the speed chosen was such that participants could still track the objects perfectly and, thus, avoid tracking errors except perhaps by attentional lapses. Task 2 differed from Task 1 since Task 2 required the same participants to keep track of the target labels. As in Pylyshyn (2004), these labels were shown alongside the targets during the target-designation phase, while no such labels were present during the tracking phase. In the response phase, participants had to click on the target and indicate its label using the number keys.

Accordingly, participants were asked to keep track of which target is which to indicate the target labels at the end of the trial. There were 15 practice trials, with 5 trials for each cases involving the number of targets as 2, 3, or 4. Participants received feedback for these practice trials indicating correct and incorrect responses. Following the practice trials, there were 30 main trials, with 10 trials for each case involving the number of targets as 2, 3, or 4. No feedback was provided for the main trials. The order of the trials in each of the practice and main blocks was randomized.

To discuss the motion dynamics of the trials, we wish to draw the readers’ attention to the shell game played by magicians. This game involves a pea or a small ball being placed under one of the three identical shells placed in a row. The shells are then exchanged rapidly and the participants may lose track of the shell under which the pea was placed originally. A magician may use tricks other than rapid motion to violate participants’ expectations of which shell contains the pea. However, we propose that rapid motion alone is sufficient for the participants’ to lose track of the shell under which the pea was placed originally. Because the shells are visually identical, the only way in which participants can keep track of the original shell is by maintaining its individuality. Following the Visual Indexing Theory based explanation of tracking, the pointer or index can easily follow the original shell and help the participant maintain its individuality. However, we propose that this is not the case, and maintaining the individuality of the original shell even as its location changes requires effort.

**Figure 8.**
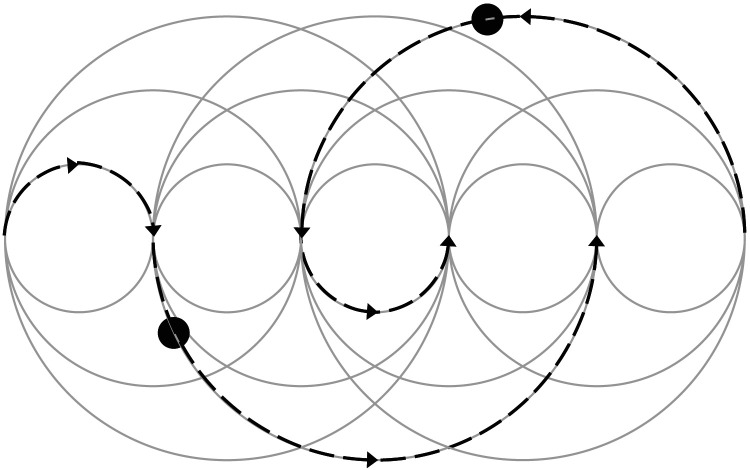
The figure illustrates the sample paths (dotted lines) of two objects (filled black circles) following motion dynamics similar to a magician’s shell game. (See the description of Task 2 under the section on Procedure of Experiment 2.) The objects were constrained to move along the edges of the circles, so that their spatial order changed rapidly. Even amongst these, only those computer generated trials were selected in which the objects always stayed at least at a distance of 120 pixels (4 degrees) from each other. With a rapidly changing spatial order, the ID maintenance mechanism as suggested in the text would have a hard time keeping a track of IDs and would tend to make errors.

In our experiment, visually identical circles replace the shells in the magicians’ game. The distinction between the shell containing the pea vs. those not containing the pea is replaced by a unique label given to each target. Then, the individuality of the different targets is maintained by the correspondence updates as discussed in the section on ‘Identity maintenance without indexes’. The resulting motion dynamics are demonstrated in Figure 8^8^.

Before the experiment, we generated trials for task 2 for each speed the calibration task could have yielded. These trials were subject to three constraints: (i) the objects followed motion dynamics similar to a magician’s shell game, (ii) no objects ever came closer than 120 pixels aka 4 degrees of visual angle in the experimental settings, (iii) our simple heuristic employed for ID tracking performed with less than 100% ID accuracy.

For the number of targets equal to 2, we generated trials for speeds ranging from 0.5 pixels per frame to 18 pixels per frame. For the number of targets equal to 3, the speeds ranged from 0.5 pixels per frame to 11 pixels per frame. For the number of targets equal to 4, the speeds ranged from 0.5 pixels per frame to 7 pixels per frame. Generating trials for higher speeds when the number of targets equaled 3 or 4 (and the number of objects equaled 4 or 5 respectively) was computationally expensive, and so was avoided.

For number of targets equal to 3 and 4, it was computationally expensive to come up with trials following the magician’s shell game dynamics that also satisfied the other two constraints; thus, we had to restrict the upper limit of the speeds in these cases to 11 and 7 pixels per frame respectively. In addition, the same computational constraints also forced us to limit the number of objects to no more than one plus the number of targets.

Thus, while the previous task had 8 objects, this task had trials containing (2 targets, 3 objects), (3 targets, 4 objects), or (4 targets, 5 objects). Each trial had 1 distractor to further rule out trials containing ID errors resulting from tracking errors due to attentional lapses.

### Results

We excluded trials in which the participant made tracking errors, assuming they resulted from attentional lapses. Out of 120 trials for each of the three cases (10 trials per participant), this resulted in the removal of

- 0 trials for the case of number of targets equal to 2.
- 3 trials (1 practice, 2 main) for the case of number of targets equal to 3.
- 12 trials (2 practice, 10 main) for the case of number of targets equal to 4.

On the remaining trials that contained no tracking errors, we conducted a Bayesian t-test using the BayesFactor library (Morey et al., 2015) for each case involving the number of targets 2, 3, or 4. The mean error rates of the participants across the practice and main trials, along with the Bayes Factor for each case are indicated in the Table 3. Bayes factor over 10 indicates that the data supports the hypothesis that ID errors are significantly different from zero, consistent with tracking not reliant on incorruptible indexes. In contrast, Bayes Factor below 0.1 indicates that the data supports the hypothesis that ID errors is not significantly different from zero, consistent with tracking based on incorruptible indexes. Anything in between indicates an ambiguous result. With these thresholds, we note that there are statistically significant ID errors for the number of targets equaling 3 or 4 even without tracking errors.

**Table 3.** Mean ID error rates of the participants across the practice and main blocks for each case of number of targets equal to 2, 3, and 4. The Bayes factor corresponding to the support for the hypothesis that ID errors are non-zero against the hypothesis that the ID errors are zero are shown with the mean error rates in brackets next to them.

Block \ Number of Targets (Bayes Factor, *BF*_10_)	2	3	4
Practice	0.44 (1.67%)	12.6 (15.8%)	147.3 (23.8%)
Main	1.32 (5.83%)	142.0 (15.4%)	20.7 (20.0%)

### Discussion

In this second experiment, we attempted to prevent index reassignments to ensure that ID errors do not take place due to index reassignments. This was enabled by restricting the separation of objects to at least 4 degrees of visual angle even at the 19 degrees of eccentricity that our experiment required. We also eliminated trials with tracking errors in expectation that they were caused due to attentional lapses. In this case, non-significant tracking-ID accuracy disparity would lend support to the indexing-based account of tracking. Non-significant results would also explain the tracking-ID accuracy disparities in Pylyshyn (2004)—the disparities resulted from index reassignments due to the close approach of objects as suggested by their post-hoc analysis.

With a Bayes Factor threshold of 10, we obtained statistically significant tracking-ID accuracy disparity for the case of three and four targets (Table 3), the latter case of four targets being consistent with results from Pylyshyn (2004) and our Experiment 1. These results support the view that multiple object tracking in humans may not require pre-attentive indexing.

However, it is important to emphasize that these results cannot be used as definitive evidence for this position. While a separation of 4 degrees is necessary to enable attentional resolution, it may not be sufficient to prevent FINSTs from losing their referents. Also, as Pylyshyn (2004) observe, the tracking-ID discrepancy is theoretically interesting only if we assume that discrete references are cognitively available outside the tracking task. It is certainly conceivable for FINSTs to only operate inside a tracking mechanism, while being inaccessible elsewhere.

## COMPUTATIONAL MODELING

### MOTUAF-Unitary: Multiple Object Tracking as Updates of Attended Features, Augmented by Unitary Cognition

So far, we have provided evidence supporting that MOT takes place without indexes and outlined how this might happen. In this section, we detail a computational model for this task and explore the implications of the conditions under which it can achieve patterns of tracking performance that have been found in the literature.

To do this, we need to be more concrete about the structure of the maps we have so far only identified conceptually. We assume that both the maps may be modeled by a two-dimensional rectangular grid of cells each indexed by Cartesian coordinates. Note that the assumption of Cartesian indexing need not be true; we are open to testing our model using a circular grid involving polar coordinates or any other more neuropsychologically plausible representations.

Next, we provide a formal treatment of the two maps, a unitary buffer, and their interactions. For the rest of this paper, let *t* denote an arbitrary point of time, and at that time point, let

1. matrix *O*^*t*^ denote the grid of objects containing their actual locations
2. matrix *A*^*t*^ denote the grid of attended locations
3. *U*^*t*^ = [*u*^*t*^, *v*^*t*^] denote a capacity-two sequence of locations corresponding to the unitary process

The notion of unitary processing will be made clear in the upcoming section.

#### The Involvement of Velocities.

**Figure 9.**
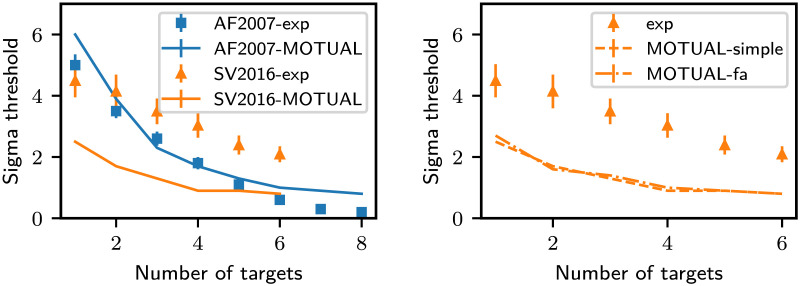
Left: The plots compare the object speeds vs. number of targets of the model against empirical data from Alvarez and Franconeri (2007) and Srivastava and Vul (2016), respectively AF2007 and SV2016, for 94% and 80% accuracy. The model uses round-robin updates of attended locations with just the retinotopic object map and attention map without any unitary processing. Right: MOTUAF-simple of the same model as the one used on the left. MOTUAF-fa is a model variant with updates of attended locations, always prioritizing the most crowded locations (see the section on Unitary Processing). The rest of the model is the same as the one used for the left plot, with just the retinotopic object map and attention map without the unitary processing. A better update sequence can increase tracking performance for cases when the number of targets exceeds 1. In this case, we obtained no performance advantage even for those cases. We wish to bring the reader’s attention to the performance disparity between the human and model performance in the single target case that calls for the use of information other than the instantaneous object locations provided through the retinotopic object location map. In both cases here, the *f*_*loc*_ of the model equaled the frame-rate of the display, with both being equal to 30 Hz.

While trying to reproduce previous results on patterns of tracking performance, we noted that relying solely on the information in a retinotopic map of instantaneous locations of objects results in tracking performance worse than humans in a particular case. This case arose when we tried to obtain the tracking performance pattern corresponding to object speed vs. the number of targets from Alvarez and Franconeri (2007) and Srivastava and Vul (2016) (Figure 9). In Alvarez and Franconeri (2007), the objects were constrained to not come closer than 4 degrees of visual angle, but no such constraints were imposed in Srivastava and Vul (2016); the objects were free to cross each other’s paths in Srivastava and Vul (2016). The speed limit corresponding to a particular accuracy of the model was almost the same as humans for Alvarez and Franconeri (2007) but was only half as humans in Srivastava and Vul (2016). The poor speed limit of the model held even when a single target was tracked. Thus, the disparity could not be attributed to a better update scheme^9^. This called for the use of additional information for tracking.

Note that the need for this additional information is not changed by changing the structure of the retinotopic maps. Indeed, changing the structure of the map of object locations and attended locations can change the structure of the required additional information. However, additional information—something more than the instantaneous locations—will still be required. We further note that the performance limitation persists despite using a grid resolution equal to the display resolution.

A natural candidate for this additional information is (locally computed) velocity information. Holcombe (2022) has outlined two possibilities for using such information. One possibility is to use velocity to estimate locations; however, several studies (Franconeri et al., 2012; Keane & Pylyshyn, 2006) have noted the use of velocity information to estimate locations as psychologically implausible.

Keane and Pylyshyn (2006) test participants’ tracking ability in the presence of occlusion. The targets disappear briefly and reappear after a short interval of time. They report that participants track better when the post-occlusion location of the targets is closer to their pre-occlusion locations, than when the post-occlusion locations are closer to their motion-extrapolated pre-occlusion locations. Franconeri et al. (2012) report similar results.

However, all the experiments in Franconeri et al. (2012) and Keane and Pylyshyn (2006) used 4 targets and 4 distractors. Howe and Holcombe (2012) report that predictable velocity information led to better tracking when there were two targets but not when there were four targets. Thus, the use of velocity information to estimate locations remains plausible in MOT.

Holcombe (2023) has suggested that human performance in multiple object tracking may be a result of two systems working together—(i) velocity-using unitary cognition, that is, a low-capacity process referred to as System 2 in the broader literature, along with (ii) a nearest-neighbour-heuristic based high-capacity low-level process. The use of velocity information for position estimation may then be the result of the involvement of System 2. With the involvement of both the systems, it becomes natural to explain why velocity information is useful when there are few targets but not when they are more. Our simulations are in agreement that incorporating both systems is sufficient for obtaining the patterns in tracking performance found in the literature.

#### The Object Location Map and the Attended Location Map.

$O_{ij}^{t}$ = 0 indicates the absence of any object in the grid cell at row *i* and column *j*, and $O_{ij}^{t}$ = *n* indicates n objects in that grid cell. Similarly, $A_{ij}^{t}$ = 0 indicates that the grid cell at row *i* and column *j* is not being attended to. Any larger value indicates that the location is being attended to with one or more objects (equal to $A_{ij}^{t}$) at that location available for reporting.

At the start of the trial, at time *t*_0_, attended locations will certainly be identical to the locations of the targets. That is, for each location where $A_{ij}^{t_{0}}$ is non-zero, $O_{ij}^{t_{0}}$ is also non-zero. As the trial progresses, the attended locations may or may not correspond to the exact locations of the objects. At an arbitrary time *t* during the trial, some $A_{ij}^{t}$ may be non-zero even though $O_{ij}^{t}$ is zero. This discrepancy between $O_{ij}^{t}$ and $A_{ij}^{t}$ is the main difference with respect to the FINST theory, which considers locations as being accessed through the indexed objects, thus disallowing attention to locations without objects. As suggested earlier while describing the outline, the idea that the position representations of targets are not always identical to targets is compatible with the findings in Howard and Holcombe (2008) and Howard et al. (2011). In light of the findings suggesting object-based attention, it may be noted that the lag between the position representations and the actual positions of the targets has been reported to be around 10–130 ms (Holcombe & Chen, 2013; Howard & Holcombe, 2008). Thus, even though attention may be object-based even in MOT, we suggest that a certain duration is required for the proto-objects to capture attention. However, the exact nature of this duration will require additional experiments.

Thus, in this framework, multiple object tracking consists of the maintenance of attended features (in our case, locations) so that they keep corresponding to objects as far as possible. Such maintenance involves an update of attended features (locations) that no longer correspond to any objects to other nearby features (locations) that correspond to some object. The update from *t* to *t* + 1 can be understood as the maintenance of the following relation between the entries in *A*^*t*^ and *A*^*t*+1^:

$$A_{ij}^{t}=1\Rightarrow \left\{\begin{array}{ll} A_{ij}^{t+1}=1 & \text{if}\;O_{ij}^{t\prime }\neq 0\\ A_{\text{unitary}‐\text{cognition}‐\text{estimate}\left(i,j\right)}^{t+1}=1 & \text{if}\;O_{ij}^{t+1}=0\;\text{and}\;u^{t+1}=\left(i,j\right)\\ A_{\text{nearest}‐\text{object}‐\text{location}\left(i,j\right)}^{t+1}=1 & \text{if}\;O_{ij}^{t+1}=0\;\text{and}\;u^{t+1}\neq \left(i,j\right) \end{array}\right.$$ (1)

Here, the condition *u*^*t*+1^ = (*i*, *j*) is true if the unitary process is processing the location. The other entries in *A*^*t*+1^ are set to 0. The above update assumes binary values of the entries in *A*^*t*^ for simplicity, but the model allows larger values of both *O* and *A* grid cells, which arise when objects overlap within grid cells.

This update is assumed to be an expensive, constrained resource process, which we characterize by a frequency of location updates *f*_*loc*_. This frequency denotes the total number of updates across all attended locations happening every second, so that greater the number of attended locations, less frequent are the updates to each location and, thus, worse is the maintenance. In the section on “MOTUAF Attention Updates”, we explain how more attentional allocation leads to greater spatial precision as reported and utilized by Alvarez and Franconeri (2007) and Srivastava and Vul (2016).

Most importantly, unlike FINSTs (Pylyshyn, 1989, 2009) and MOT models based on FINSTs that enable individuating objects without considering their visual features or even their locations, all attended locations in our model—except the one processed by the unitary system—are indistinguishable from each other except by their locations themselves.

Further, following Alvarez and Franconeri (2007), we assume that the total number of attended locations can vary within an individual based on task requirements. We expect an upper limit to the number of attended locations; it is certainly above 4, given the evidence illustrating that subjects can track as many as 8 objects moving at low velocities (Alvarez & Franconeri, 2007). We speculate that the limit should be related to one’s working memory capacity but leave the exact number open as a question for future work to address.

Note that there are two kinds of updates in the model. The first kind involves updates of the unitary processing, including deciding which attended object should be processed using the unitary system. The second kind involves the updates to attended locations, which correspond to the actual MOT task. We discuss each of these in the following two subsections.

#### Unitary Processing.

In order to use velocity information to estimate the location of an object, we need information about its location at at least two points in time. Following Holcombe (2022, 2023), we restrict our model to do this only for a single object at any time step. So, whenever a different object needs to be processed by the unitary system, the unitary processing’s existing information will be discarded. Then, the location information corresponding to the new object will be noted.

We propose that unitary processing is utilized for targets in the greatest danger of being confused with distractors. This proposal is consistent with the counter-intuitive finding in Srivastava and Vul (2016) that crowded locations are tracked better.

For purposes of notation, let the subscripts denote one particular location on the retinotopic map, and let the superscripts denote the particular instance of time for which these are being considered. According to this notation, $a_{1}^{t}$, $a_{2}^{t}$, …, $a_{n}^{t}$ will be the non-zero grids in *A*^*t*^ at time point *t*, with *n* being the number of targets.

Then, for the attended location $a_{i}^{t}$, the confusion is quantified by a confusion-ratio given by:

$$c_{i}^{t}={\left\{\frac{\text{dist}\left(a_{i}^{t},\text{nearest}‐\text{object}‐{\text{location}}^{*}\left(a_{i}^{t},1\right)\right)}{\text{dist}\left(a_{i}^{t},\text{nearest}‐\text{object}‐{\text{location}}^{*}\left(a_{i}^{t},2\right)\right)}\right\}}^{c_{e}}$$ (2)

Here, nearest-object-location* is a slight variant over the nearest-object-location mentioned previously.

- nearest-object-location takes in only the location *l* from where to start the search and thus returns the location of the object *l*′ closest to that location *l*
- nearest-object-location* takes in the location *l* as well as a number *k* and returns the location of the *k*th closest object nearest to the location *l*

Thus, for *c*_*e*_ > 0, the confusion-ratio expresses that the confusion will be the greatest when the distance between the second closest object and the attended location is as close to the distance between the first closest object and the same attended location. In other words, a high confusion-ratio indicates that the second closest object might come closer to the attended location than the (first) closest object. Thus, the two may be confused. Note that the confusion ratio is computable using information locally available for each attended location.

The updates of attended locations then proceed in a manner determined probabilistically by the confusion ratios. This probability is given by

$$p_{i}^{t}=\frac{c_{i}^{t}}{\sum_{j=1}^{n}c_{j}^{t}}$$ (3)

Note that the greater the value of *c*_*e*_ in Equation 2, the greater the probability that a more confusable location will be chosen for update compared to a less confusable location. In particular, when *c*_*e*_ → ∞, the probability of choosing the most confusable location will be 1. In the section on ‘Reproducing earlier MOT results’, we fix *c*_*e*_ with a large value, indicating that only the most confusable locations are always chosen.

We also impose the additional restriction that once the unitary system has chosen an attended location for processing, it cannot process another attended location for about 100 to 300 ms. In other words, we fix the frequency *f*_*u*_ at which the unitary system can choose which attended location to process at about 3–10 Hz. Our simulations suggested that this interacts with the rate at which object velocities change in the environment. This effect is consistent with the results in Howe and Holcombe (2012) that tracking accuracy is better in the *n* = 2 targets case when velocities are predictable. They do not find the benefit of predictable velocities for tracking accuracy in the *n* = 4 targets case. Unfortunately, *n* = 2 and *n* = 4 are the only two cases they considered. Furthermore, they do not vary the predictability of velocities. Our model predicts that the longer the velocity information of a particular target is usable, the more beneficial is the unitary processing. Suppose the velocity information changes faster than the rate at which unitary processing can choose which attended location to process. In that case, the velocity information will not be helpful.

The unitary processing allows the computation of velocity through the positions of the objects at two points in time. This information is then used to estimate the next location whenever the location being updated is the same as the location that is a part of the unitary processing. A attended location (*i*, *j*) is part of the unitary processing if *u*^*t*+1^ = (*i*, *j*) (see Equation 1). In this case, the nearest-object-location search begins from that location. For other attended locations that are not part of the unitary processing, only a simple nearest-object-location is used starting from the attended location itself, without relying on any velocity information. The nearest-object-location algorithm is elaborated in the next section.

Overall, with the unitary processing, whenever the location that is being updated is the same as the location that is a part of the unitary processing (indicated by the condition *u*^*t*+1^ = (*i*, *j*) in Equation 1), then because the unitary processing allows the computation of velocity through the positions of the objects at two time points, this information is then used to estimate the next location and the nearest-object-location search begins from that location. For other attended locations which are not a part of the unitary processing, only a simple nearest-object-location is used starting at that attended location itself without relying on any velocity information. This process is elaborated in the next section.

#### MOTUAF Attention Updates.

We assume sequential updates for each of the attended locations in a manner determined by their crowding. According to the discussion in the previous section, this means that the most crowded location gets the update. This update may or may not happen due to the unitary process itself.

Recall that Pylyshyn and Storm (1988) had shown that a serial tracking algorithm based on a spotlight of attention moving between the objects at finite speeds could not account for the tracking accuracy on the task of tracking multiple identical objects. But, they did not rule out the case of quantal updates. Egeth and Yantis (1997) provides evidence that the movement of attention is quantal rather than analog, as well as that the RTs involved in the supposedly-parallel processing of multiple stimuli are additive rather than subadditive (and therefore the supposition of parallel processing is unwarranted). Serial switching theory also naturally accounts for variation in temporal resolution of tracking with the number of targets (Holcombe & Chen, 2013), as well as the increase in temporal lags in tracking with the number of targets (Howard & Holcombe, 2008).

Thus, in every time step, only a single attended location is updated. So, the greater the number of locations that need to be attended, the less frequent the updates to each location; thereby, the tracking accuracy with an increasing number of targets is worse.

1. We note that an attended location $a_{i}^{t}$ corresponds to the exact location of some object only immediately after an update at time *t* caused it to be non-zero.
2. The frequency of updates of the attended locations is denoted by the parameter *f*_*loc*_. Suppose the next update corresponding to $a_{i}^{t\prime }=a_{i}^{t}$ happens at time *t*′, so that $a_{i}^{t\prime +1}$ once again corresponds to some object.
3. At time *t*′, the attended location $a_{i}^{t\prime }=a_{i}^{t}$ may no longer correspond to an object, since during the time from *t* to *t*′, the object would have moved from the location $a_{i}^{t}$ to a new location *l*. While updating $a_{i}^{t\prime }=a_{i}^{t}$ to $a_{i}^{t\prime +1}$ at time *t*′,
  (a)  Suppose $a_{i}^{t\prime }$ is different from the location in the unitary processing; that is, suppose $a_{i}^{t\prime }\neq u^{t\prime }$. Then the model finds the object nearest to $a_{i}^{t\prime }$. To do so, it looks for a location occupied by *some* object with increasing distance from $a_{i}^{t\prime }$; thus, it avoids considering all the objects on display to find the nearest object. This local search is also characterized by another parameter called the nearest object bound *nob* since it is unreasonable to assume that recovering the object could work if they have moved too far away from the attended locations. The search is aborted, and the location is no longer stored if no object is found within a distance of *nob* from $a_{i}^{t\prime }$.
  (b)  On the other hand, if $a_{i}^{t\prime }=u^{t\prime }$, then instead of finding the nearest object in the vicinity of $a_{i}^{t\prime }$, the model finds the nearest object in the vicinity of $a_{i}^{t\prime }+\left(u^{t\prime }-v^{t\prime }\right)$ strictly following the method described in the previous sub-step.
4. Suppose this new location where *some* object is present is *l*′. Then, the update is performed so that $a_{i}^{t\prime +1}$ = *l*′ holds. In general, *l*′ may not be the same as *l*. Suppose the object has moved predictably (unitary system processing) or has not moved much (non-unitary system processing). In that case, *l*′ will more likely be the same as *l*, and in these cases the model will not lose track of the target; but otherwise, the locations *l* and *l*′ will be different and correspond to different objects. According to our model then, this is how tracking errors occur.

### Reproducing Earlier MOT Results

As Srivastava and Vul (2016) point out, explaining the degradation of accuracy with an increase in the number of targets is the stiffest challenge for computational MOT models. In this section, we show how MOTUAF successfully reproduces this trend across *in silico* reproductions of four different experiments, PS1988 (Pylyshyn & Storm, 1988), AF2007 (Alvarez & Franconeri, 2007), FR2008 (Franconeri et al., 2008) and SV2016 (Srivastava & Vul, 2016).

#### Methods.

In our simulations, the size of the retinotopic grid map of the model equals the size of the MOT window on the display (in pixels). Indeed, the amount of information is limited by the MOT window—in terms of its resolution and the refresh rate; any model based on a retinotopic grid cannot use a resolution exceeding that of the display.

We use two kinds of dynamics depending on the characteristics of the experiments: constant speed dynamics and Ornstein-Uhlenbeck dynamics. For each of the two dynamics, we also outline the relationship between the parameters usually reported in the MOT literature and those required for simulating an equivalent environment in a computer program. We achieve this by equating the average duration an object takes to travel from one end of the MOT window to another in the experimental setting and our simulation. In both cases, the only information the model receives is a grid map indicating the instantaneous locations of the objects. It does not have any direct access to the velocity information or the correspondence information.

##### Constant Speed Dynamics.

The first kind of dynamic is based on Alvarez and Franconeri (2007) wherein objects move at constant speeds, repelling each other if they come within a certain minimum distance of each other or the walls. Similar kinds of dynamics have been stated in Franconeri et al. (2008). In particular, to identify the dynamics of a particular object for the next time step, we identify all the objects and walls that are within a certain minimum distance from that object. For each such wall or object, we perform a component-wise (x-component and y-component) sum of the inverse-square distances to determine the net direction of repulsion. This net direction of repulsion forms the direction of the object at the next time step. That is, for an object at (*x*_*i*_, *y*_*i*_) moving at speed *σ* in direction *θ* (angle with respect to the x-axis), its new direction of motion is given by

$${rx}_{net}=\sum_{j\neq i}\frac{x_{i}-x_{j}}{\sqrt{{\left(x_{i}-x_{j}\right)}^{2}+{\left(y_{i}-y_{j}\right)}^{2}}}$$

$${ry}_{net}=\sum_{j\neq i}\frac{y_{i}-y_{j}}{\sqrt{{\left(x_{i}-x_{j}\right)}^{2}+{\left(y_{i}-y_{j}\right)}^{2}}}$$

$$\theta^{\prime }=\arctan\left({ry}_{net},{rx}_{net}\right)$$

where *j* varies over all the objects and walls within a certain minimum distance of the object at (*x*_*i*_, *y*_*i*_). For walls, we consider the perpendicular distance from the wall.

By equating the time it takes for an object to cross from one side of the display to another in the experiment vs. the simulation, we obtain

$$\frac{\theta }{d}=\frac{D}{\eta \sigma \frac{D}{s}}$$

Here,

- *θ* degrees is the angle subtended by the MOT window
- *d* degrees per second is the average speed of the object
- *D* is the actual width of the grid on the display
- *η* is the number of simulation updates to be carried out per second
- *σ* is the speed of the object in pixels per second in the simulation
- *s* is the side of the grid (in pixels)

Solving for *σ*, one obtains

$$\sigma =\frac{s\cdot d}{\eta \cdot \theta }$$ (4)

##### Ornstein-Uhlenbeck Dynamics.

The second kind of dynamics is from Srivastava and Vul (2016) and Vul et al. (2009). In this, objects move according to Ornstein-Uhlenbeck dynamics with

$$x_{t}=x_{t-1}+v_{t}$$

$$v_{t}=\lambda v_{t-1}-{kx}_{t-1}+w_{t}$$

$$w_{t}∼N\left(0,\sigma \right)$$

The y components are given similarly. Following the same notation as Vul et al. (2009), we set the spring constant parameter to *k* = 0.0005 and the inertia parameter *λ* = 0.9 unless stated otherwise. The motion dynamics described in Pylyshyn and Storm (1988) involves (i) a change in speed and direction of the objects every few hundred milliseconds (ii) a restriction on objects to always stay a certain minimum distance away from each other. We achieve similar dynamics by relying on the Ornstein-Uhlenbeck dynamics with an additional constraint. This constraint lets objects change velocities as required by the Ornstein-Uhlenbeck dynamics. However, their positions change only if the objects remain a certain minimum distance away from each other after the change.

The relation between the empirical and simulation parameters is similar to the one obtained for constant speed dynamics, but with a factor of 1.8 thrown in. We noted that for *k* = 0.0005 and *λ* = 0.9, one update following unconstrained Ornstein-Uhlenbeck dynamics covered an average of 1.8*σ* pixels. Thus, we have^10^:

$$\frac{\theta }{d}=\frac{D}{1.8\eta \sigma \frac{D}{s}}$$

Solving for *σ*, one obtains

$$\sigma =0.555\times \frac{s\cdot d}{\eta \cdot \theta }$$ (5)

#### Results.

For purposes of tracking, *f*_*loc*_, *nob*, *c*_*e*_, and *f*_*u*_ constitute the free parameters of the model. The free parameters of the environment and simulations include the grid size, *σ*, MOT simulation update rate, and the total number of simulation updates carried out (or equivalently, the number of time steps).

**Figure 10.**
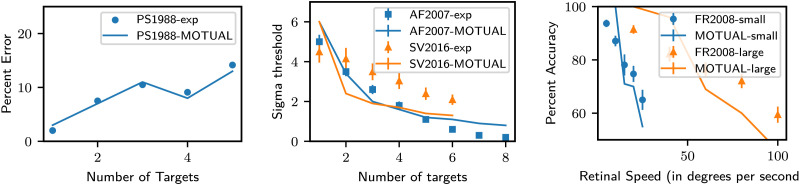
Reproducing previous results in Multiple Object Tracking. Left: Comparison of percentage errors with increasing number of targets corresponding with a fixed average speed of MOTUAF plotted against data from Pylyshyn and Storm (1988). Center: Velocity threshold vs. number of targets patterns for MOTUAF plotted against data from AF2007 (Alvarez & Franconeri, 2007) and NS2016 (Srivastava & Vul, 2016). Right: Comparing our model against the human data in experiment 1 of Franconeri et al. (2008).

Figure 10 summarizes MOTUAF’s behavior vis-a-vis data from all the four experiments we evaluated. Table 4 enumerates the experiment-specific parameters and free parameter values used to produce these results. MOTUAF, with only three free parameters, successfully reproduces the trends seen in all four experiments.

**Table 4.** MOTUAF simulation parameters corresponding to data from previous empirical results.

Parameters \ Paper	PS1988	AF2007	SV2016	FR2008-small	FR2008-large
Visual Angle of MOT Window	21.5°	30° × 24°	16°	20.5° × 9.1°	82° × 36.4°
–display resolution	not given	not given	720 × 720	175 × 078	700 × 310
–grid-size for MOTUAF	360 × 360	720 × 720	720 × 720	180 × 180	720 × 720

Retinal Speed	1.25–9.4°/s	0.1–16°/s	not given in °/sec	5–25°/sec	20–100°/sec
Trial Duration	10 sec	5 sec	5 sec	6 sec	6 sec
Intervals at which velocities or directions change	“few hundred ms”	–	–	0.3°/frame	0.3°/frame
Number of updates	25	300	150	540	540
Environment Kind	Ornstein-Uhlenbeck	constant-speed	Ornstein-Uhlenbeck	constant-speed	constant-speed
Sigma for simulations	18	0.1–6.0	1.3–5.4	0.45–2.25	1.80–9.0

Minimum Object Distance	15 (0.75°)	80 (4°)	0 (0°)	24 (2.8°)	96 (11.3°)

MOTUAF Free Parameters					
–*f*_*u*_	10 Hz	3 Hz	10 Hz	10 Hz	10 Hz
–*c*_*e*_	∞	∞	∞	∞	∞
–*f*_*loc*_	28 Hz	30 Hz	30 Hz	17 Hz	16 Hz
–*nob*	∞	24	60	∞	∞

##### Errors vs. Number of Targets for a Particular Average Speed.

Empirical research into multiple object tracking began with Pylyshyn and Storm (1988). They asked participants to detect flashes occurring on target objects and measured the error rates of this detection. A successful detection of the flash indicated successful tracking, while a failed detection indicated an error in tracking. In Figure 10 (left), we reproduce the pattern of percentage error rates for tracking the number of targets varying from 1 to 5, as measured by Pylyshyn and Storm (1988). This uses the Ornstein-Uhlenbeck dynamics with an additional constraint that objects never get close to each other. The detailed experimental and simulation parameter values are given in Table 4.

##### Object Speed Thresholds for a Particular Accuracy vs. Number of Targets.

In Alvarez and Franconeri (2007) and Srivastava and Vul (2016), the authors varied the speed of the objects to find the maximum speed at which the tracking accuracy of the subjects reached a predetermined threshold. This calibration was done for the number of targets varying from 1 to 8 in Alvarez and Franconeri (2007) and 1 to 6 in Srivastava and Vul (2016). In their tasks, a target or a distractor became visually distinctive with equal probability at the end of a trial. Subjects had to indicate whether this visually distinctive object was a target or a distractor. The accuracy of these responses across many trials was dubbed as tracking accuracy. Our model attempts to reproduce these patterns in Figure 10 (center). The model’s speed threshold is close to humans for the data from Alvarez and Franconeri (2007), who always kept objects a certain minimum distance away from each other. But, the model’s speed threshold could not reach human performance for 2 or more targets for the data from Srivastava and Vul (2016), who allowed objects to overlap. Investigating this discrepancy requires more empirical work.

##### Tracking Accuracy vs. Object Speed in Scene-widths Per Second.

One might expect that, when object crowding is controlled, tracking capacities would depend on the objective speed of the objects or at least on the angular speed of the objects at the retina. In contrast, Franconeri et al. (2008) present an interesting finding that the tracking accuracies for the same object speed on small and large displays are very similar when the object speed is measured in scene-widths per second. An object speed of 1 scene width per second means that if the object were allowed to move freely without changing direction, it would move from one end of the MOT window to another once in 1 second. A speed of 1.25 scene widths means that the object will cover 1.25 times the width of the MOT window in 1 second; a speed of 0.5 scene widths means it will cover 0.5 times the width. The small display subtended an angle of 20.5° × 9.1° at the retina, while the large display subtended an angle of 82° × 36.4°. Figure 10 (right) shows our model reproducing these patterns. Nevertheless, the minimum distance between the objects scaled with the display size, and their findings are consistent with the attentional resolution capacities measured by Intriligator and Cavanagh (2001).

### Multiple Identity Tracking With MOTUAF

Given that at time *t*, the system only has access to the locations $a_{1}^{t},\ldots ,a_{n}^{t}$ but not the locations at other points of time, the information it has so far is insufficient to make conclusions about the target IDs.

To keep track of IDs, we therefore propose that at time *t*, there also exists a separate sequence $q_{1}^{t},\ldots ,q_{n}^{t}$ of IDs. One possible strategy to maintain such a sequence is to keep reciting the sequence. To match objects to IDs, one can go over the attended objects in some spatial sequence while reciting their IDs in that same sequence.

One particular sort order (but not the only one) could be to sort the locations in the non-decreasing order of x-and-y coordinates. With this, at the start of the trial at time *t*_0_, the sequence of attended locations is monotonic in their x-and-y coordinates, so that for *m* < *n*, $a_{m}^{t_{0}}\equiv \left(x_{m}^{t_{0}},y_{m}^{t_{0}}\right)$ and $a_{n}^{t_{0}}\equiv \left(x_{n}^{t_{0}},y_{n}^{t_{0}}\right)$ are such that ($x_{m}^{t_{0}}<x_{n}^{t_{0}}$) or ($x_{m}^{t_{0}}=x_{n}^{t_{0}}$ and $y_{m}^{t_{0}}\leq y_{n}^{t_{0}}$). Given this order of $a_{1}^{t_{0}},\ldots ,a_{n}^{t_{0}}$, the system now has the ID of the object at $a_{k}^{t_{0}}$ in $q_{k}^{t_{0}}$ for *k* ∈ 1, …, *n*, and it is through this correspondence that the system can infer the IDs of the objects.

At the end of the trial at time *t*_*e*_, the system again sorts the sequence of attended locations $a_{1}^{t_{e}},\ldots ,a_{n}^{t_{e}}$ using the same sorting-rule that it had used at the start of the trial, in our particular example, this is non-decreasing order of x-and-y coordinates. It then assigns the ID $q_{k}^{t_{e}}$ to the object that is at or nearest the location $a_{k}^{t_{e}}$.

The strong assumption here is that the sequence of IDs is *never* updated. In the general case, and as one’s intuition would suggest, it should be possible to update the ID sequence, aka do a “correspondence update”, especially if the objects move sufficiently slowly. This update can be characterized by an additional parameter for the model, which we call the frequency of correspondence updates *f*_*corr*_. The question of whether or not a correspondence update takes place at any particular time step is relevant only if a location update has taken place. So, as opposed to the frequency of location updates *f*_*loc*_ being an absolute frequency, *f*_*corr*_ can be understood as a frequency relative to *f*_*loc*_. Thus, 0 ≤ *f*_*corr*_ ≤ 1.

**Figure 11.**
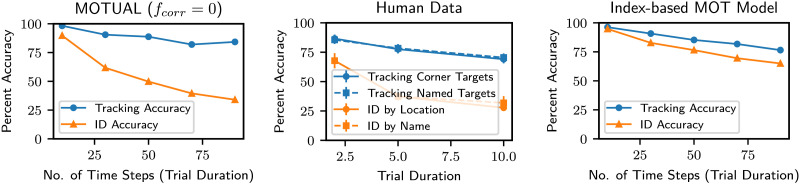
Tracking and ID accuracy in an MOT task with 4 targets and 8 objects (4 targets, *σ* = 1.25) with increasing trial duration. Left: Predictions from our modeling assuming *f*_*corr*_ = 0. Center: Replotted results from Pylyshyn (2004). Right: Predictions from a model based on indexes aka *f*_*corr*_ = 1. Our model only captures the notion of an ID without distinguishing between Corners vs. Names.

When *f*_*corr*_ = 1, that is, when correspondence updates occur whenever location updates occur, MOTUAF (like FINST-based models) predicts ID accuracy being identical to tracking accuracy across any trial duration (see Figure 11, right). Setting *f*_*corr*_ to less extreme values produces a disparity between tracking and ID accuracy, as anticipated in the literature (Pylyshyn, 2004). For example, *f*_*corr*_ = 0 reproduces the empirical results seen in Pylyshyn (2004) (see Figure 11, center) very precisely (see Figure 11, left).

### Model-based Analysis of Experiment 1

We minimized the mean-square error (MSE) between the tracking accuracy of humans and MOTUAF for *f*_*loc*_ ranging from 6 to 45Hz, and for *nob* varying from 20 to 60.

**Figure 12.**
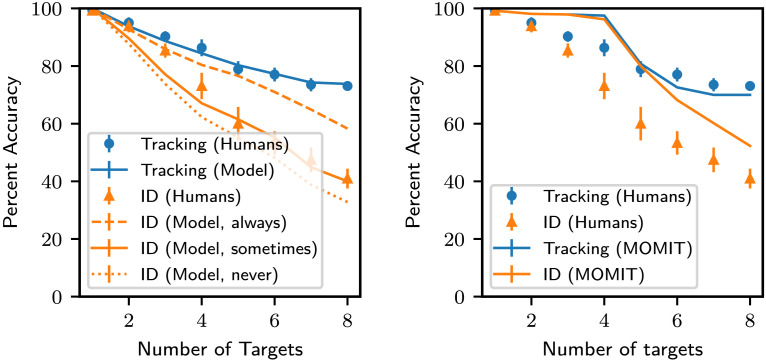
Left: Comparison of MOTUAF’s tracking and ID accuracy against human data after fitting MOTUAF’s tracking accuracy to the human data, with differing assumptions about the ID updates (i) ID updates taking place always whenever location updates take place (ii) ID updates taking place some of the times (iii) no ID updates taking place ever as with the Center figure. Error bars represent 1 SEM. Right: Comparison of MOMIT’s Tracking and ID accuracy against human data for the lowest MSE run.

For lowest MSE (Figure 12, left), *f*_*loc*_ = 30 Hz, *nob* = 52, *MSE* = 2.0 × 10^−4^, *r*^2^ = 0.980.

Comparing the model’s ID accuracy with human ID accuracy (Figure 12, left-dotted), one notes that *f*_*corr*_ = 0 results in the model’s ID accuracy being worse than the human ID accuracy (*MSE* = 5.8 × 10^−3^, *r*^2^ = 0.975). Calculating the MSE scores between the model’s ID accuracy and human ID accuracy for different *f*_*corr*_ yielded a lowest MSE for *f*_*corr*_ = 0.4 (*MSE* = 1.6 × 10^−3^, *r*^2^ = 0.971) (Figure 12, left-solid).

However, for the particular correspondence-update heuristic we employed, even with *f*_*corr*_ = 0.4, the human ID accuracy consistently exceeds the model’s ID accuracy when the number of targets is at most four. Investigating the existence, characteristics, and the disruption of the correspondence-update heuristic will require more work.

We also compare MOTUAF’s performance against MOMIT (Oksama & Hyönä, 2008), an index-based model designed for tracking visually distinct objects; however, it can track visually identical objects by suppressing its corrective attention shift mechanism. Figure 12 (right) shows MOMIT’s fit to our data. Despite a still-reasonable (*r*^2^ = 0.877) fit for tracking accuracy, we note that MOMIT is intrinsically predisposed to align ID accuracy with tracking accuracy as with the *f*_*corr*_ = 1 case (see Figure 12, left-dashed).

### Predictions and Limitations

The computational model of MOTUAF we discussed successfully replicated several empirical results. However, it required certain assumptions, and these assumptions make testable predictions. In addition, some more phenomena are still left unexplained. Below, we discuss both these issues.

#### Velocity-based Constraints.

Howe and Holcombe (2012) presented a nuanced finding that velocity information is used for tracking in the *n* = 2 targets case but not in the *n* = 4 targets case. Our modeling work builds upon this and suggests the limits under which velocity information can be used. This can be explored further. In particular, let *f*_*v*_ be the frequency at which velocity changes and $f_{v}^{*}$ be the limiting frequency at which velocity information still benefits tracking. Then, our model predicts that $f_{v}^{*}$ should decrease with an increase in the number of targets. This can be tested.

In trying to reproduce the results from Srivastava and Vul (2016) (Figure 10, center), even though we were successful in achieving human-level performance in the single target case, our model still falls short of human performance in cases involving more than a single target. Moreover, the pattern of results (a linearly decreasing trend) is quite distinct from both the model predictions as well as from the ones in Alvarez and Franconeri (2007) (a logarithmically decreasing trend). Whether this is an experimental artifact, a result of better response strategy^11^, a limitation of the model, or something else remains to be identified.

In light of the results in Franconeri et al. (2012), Howe and Holcombe (2012), and Keane and Pylyshyn (2006) discussed in the section on “The involvement of velocities” while describing the computational model MOTUAF, there indeed are strong constraints to the use of velocity information in the case of multiple targets.

#### Temporal Constraints.

Verstraten et al. (2000) noted a temporal limit of about 5–8 Hz on attentional tracking. A similar limit of 7 Hz has been noted by Holcombe and Chen (2013) for the single target case. However, our model makes no space for accounting for these. The limit predicted by our model using *f*_*loc*_ is as high as 30 Hz.

Given the phi phenomena (Wertheimer, 1912), certainly, some visual processes occur at this high a frequency. What, then, could explain how the 5–8 Hz frequencies arise?

One way to explain this could be to analyze how errors can arise in the tasks employed in Holcombe and Chen (2013). In these tasks, tracking is achieved through attention, and thus, targets are what one attends to; one notes that one would make an error if the distractor appears too soon after the target. Suppose the distractor appears too soon after the target. In that case, attention captures the distractor too. We propose that this unwarranted capture is the cause of tracking errors. Specifically, an error occurs if the distractor appears within 1/f seconds after the target, with the tracking limit being f Hz.

These results can be explained if tracking can be mechanized as follows: Targets are what one attends to. Once a target has moved (changed its property), attention gets redeployed to capture these changed features of the objects. These changed features serve as exogenous cues for attentional deployment. According to our computational modeling work, *f*_*loc*_ corresponds to this rate of attentional deployment, expected to be as high as 30 Hz. However, alongside the deployment of attention to the new feature, attention also gets disengaged from the previous feature. Suppose a proto-object with the not-yet-disengaged feature were to appear before the attention is disengaged. In that case, that proto-object will likely be captured by attention, leading to tracking errors. If, on the other hand, the proto-object appears after attention has been disengaged, say, during the attentional blink, then there should not be any tracking errors.

The attentional blink period has been suggested to last from 200 to 500 ms after the first target (Shapiro et al., 1997). While tracking a single target, if a distractor appears at the target location during the attentional blink, then tracking errors should not occur. In contrast, if the distractor were to appear at the target location during lag-1 sparing, then a tracking error will occur.

However, while this can explain the *n* = 1 target case, as suggested by Holcombe and Chen (2013), investigating how these phenomena vary for multiple locations remains an open question.

#### A Bimodal Distribution of Object Position Lags.

Howard and Holcombe (2008) and Howard et al. (2011) reported a dissociation between the reported locations of the objects and the actual object locations, with the former lagging behind the latter. We have assumed the involvement of a unitary system in tracking. The lag between the represented object locations (attended location) and the actual object locations should be close to zero if the update of the attended location took place via the unitary system. For the other attended locations, the lags should increase with an increase in the number of targets. Thus, we expect a bimodal distribution of lags: one set of lags close to zero, and another set of lags which increase with the increase in the number of targets.

#### The Most Crowded Locations Are Tracked Better But the Second Most Crowded Locations Are Not.

Srivastava and Vul (2016) reported a counter-intuitive finding that crowded locations are tracked better. We predict that the better tracking of the crowded locations is due to the involvement of the unitary system. However, the unitary system, by definition, can only update a single location. If other locations crowd simultaneously, then it will not be tracked better than the non-crowded location. More specifically, we expect that the second most crowded locations will not be tracked better than the non-crowded locations.

#### A Time Lag for Attention to Become Object-based That Increases With Increasing Number of Objects.

Building on the findings from Howard and Holcombe (2008) and Howard et al. (2011), suppose that the mechanism by which attended locations follow the objects is the same as object-based attention. In that case, we predict: (1) There is a time lag for attention to become object based. (2) This lag increases with an increasing number of attended objects.

## IMPLICATIONS FOR VISUAL INDEXING THEORY EXPLANATIONS FOR MOT

An index-less approach to tracking an object uses representations^12^ of the object being tracked (Figure 1). A part of the problem with the index-based account is that it tends to push the exact ways by which tracking takes place into the background by formulating tracking as a primitive process. Indeed, in the general case, tracking a visual element by relying on its representation is hard. There is no denying how effortless we find tracking in many cases. Yet, just because we find it effortless, there is no reason for the task to be done “simply”—the history of artificial intelligence is replete with problems that we once thought were easy but which, upon investigation, have demonstrated themselves to be hard^13^.

The properties of an object may change without a change in its identity (e.g., “It’s a bird, it’s a plane … no, it’s Superman!”). An indexing-based account relies on indexes as the non-conceptual, aka property-less pointers to each such object. Our account, that does not use indexes, has to rely on the properties of the objects. The criticality, however, lies in what kinds of properties are used. Our account relies on the existence of their being *some* property (conceptual or non-conceptual) that can help track the object at each time step. Each time step may have a different property that gets used for tracking.

Pylyshyn (2000) (and also philosophers like Perry (1979)) have suggested that picking out objects in our environments needs to be sometimes based on non-conceptual representations. Our proposal does not contradict the role of indexicals and demonstrative references in the taking of an action. Our work, however, does suggest that indexicals may not continue to be bound to their referents even as the properties of the referents change over time. If the properties of the referents change, a cognitive process must update that link—and not just a visual or auditory or a modality-specific process. The index-based explanation for MOT requires that the indexes remain bound to their referents over some variation in the properties of the referents. We suggest that the amount of change in the referents is much smaller than the amount of change that a tracking task involves. If this is the case, then Visual Indexing Theory can naturally continue to, for instance, explain the phenomena of subitizing (Trick & Pylyshyn, 1994) without any effect from our work.

## CONCLUSION

This paper investigates the theoretical position that indexes are not necessary for multiple object tracking. To this end, we conducted two experiments extending the observations made by Pylyshyn (2004) about the dissociation between tracking and ID accuracy.

We also computationally investigated a model of index-free multiple object tracking built according to this outline, and found that at least one index is necessary for such tracking. By reproducing a number of previous empirical results with this model, we also showed that a single index is sufficient for multiple object tracking.

Subject to the expectation that enabling attentional resolution of the objects would substantially prevent index-reassignments and enable FINSTs to remain bound to their referents, our empirical results are consistent with the existence of no more than 2 incorruptible indexes and even 4–5 corruptible indexes. However, we agree with Scholl (2009) that assuming indexing to be corruptible deprives indexes of the critical capacity they were supposed to provide: providing a correspondence link between elements at the current point of time and the elements at the previous point of time. Future experiments should test whether attentional resolution is indeed sufficient for FINSTs to remain bound to their referents. In addition, they will also need to incorporate the suggestion made by Pylyshyn (2004) that it may be the case that indexes and discrete references might not be available for use outside the tracking task which is a requirement to associate numeric labels with the targets.

The empirical success of MOTUAF demonstrates that it is possible to explain human MOT behavior without requiring the use of incorruptible pre-attentive indexes. Naturally, just because such an explanation is possible, does not mean that it is true. We note, though, that historically speaking, pre-attentive indexes were theoretically necessitated because of the assumption that solving the correspondence problem is a prerequisite to performing the MOT task (Luo et al., 2021; Pylyshyn, 2004). The theoretical novelty of our project is that we show that MOT is possible without computationally maintaining one index per object and thereby explicitly solving the correspondence problem.

In light of the discrepancy observed in our model-based analysis, where we found that fewer targets than 4 were ID’d better by humans than MOTUAF, it would be premature to conclude that FINSTs play no role in MOT. It may well be that fewer targets than 4 (Pylyshyn’s estimate of FINST count) could be tracked using FINSTs, and larger number of targets tracked by the MOTUAF mechanism. It is also possible that MOTUAF practically reduces to FINSTs while tracking fewer targets. Investigating this relationship presents an exciting avenue for future work.

We conclude by pointing out that, the MOTUAF computational model, irrespective of its psychological veridicality, opens up the possibility of producing fast multiple object tracking algorithms scalable to large sets of objects, particularly in combination with neuromorphic vision sensors (Pantho et al., 2018; van De Burgt et al., 2018). Consideration of such practical applications is also an interesting direction for future work.

## DATA AVAILABILITY STATEMENT

The data and code for the experiments and computational modeling may be found at https://osf.io/nzs9f/.

## Notes

^1^ Throughout the paper, the terms FINST, pointer, and index have been used interchangeably as a noun. The term “index” is also used as a verb in several places.^2^ In our experiments, as well as the experiment 4 of Pylyshyn (2004) relevant to our work, we ask participants to identify all the targets and label all of them. In this case, the tracking accuracy is given by *k*/*n*. However, in some other experiments in the literature, at the end of the MOT trial, one of the object becomes visually distinctive. This object is a target or a distractor with equal probability. Following this, the participant is asked to indicate whether the object is a target or a distractor. In this case, the participant has a *k*/*n* probability of identifying the target correctly if the object is a target and a (*n* − *k*)/*n* probability of identifying the distractor *incorrectly* if it is a distractor—since, according to the participant, the remaining (*n* − *k*) objects are also targets. In other words, the participant also has a *k*/*n* probability of identifying the distractor incorrectly. Overall, the average accuracy in this case boils down to 1/2 * (*k*/*n*) + 1/2 * (*k*/*n*) = *k*/*n*.^3^ Note that the model described by Oksama and Hyönä (2008) concerns the tracking of visually distinct objects. However, it is trivial to adapt it to the task of tracking visually identical objects by letting go of the corrective attention shift involved in step 4b described under its functional description.^4^ Code for computational modeling as well as experiments is available at https://github.com/digikar99/mot-wo-correspondence.^5^ A preliminary version of this work has appeared in the Proceedings of CogSci 2023 (Ayare & Srivastava, 2023). The current work extends the preliminary model to accommodate a unitary system, and presents additional experiments and analysis in support of our conclusions.^6^ While representations might refer to any information bearing state, and thus, the early visual system might represent information in *some* sense of the term ‘representation’, Pylyshyn (2007) (chapter 3) argues that there are at least two senses of the term. The first sense, referring to any information-bearing state, is better referred to as registration. A registration is necessarily causally connected to the world and there is no possibility of misrepresentation. The second sense refers to states that have a possibility of misrepresentation. These have a representational content for the organism, thereby determining the organism’s behavior. Above, when we say that the location information is not represented for index-based tracking, we have used the second sense of ‘representation’. In an index-based account of tracking, the location information is only registered and not necessarily encoded (represented).^7^ The location information of the objects present in the early visual system is necessarily non-conceptual and inaccessible to the cognitive system. Further processing may make this information accessible to the cognitive system. The information may be conceptual or non-conceptual at that point—we do not comment on it.^8^ See https://www.youtube.com/watch?v=vS0OsvXbrlg for the dynamic stimulus.^9^ By default, our attention updates followed a round-robin scheme in the case of multiple targets. A better scheme would involve updating the attended locations on a priority basis, for example, a more crowded location might get more priority for update. The notion of an update scheme is better understood through the discussion in the section on MOTUAF Attention Updates.^10^ Note that the *σ* in the two dynamics mean slightly different things. In constant speed dynamics, *σ* is the constant speed in pixels per second at which the object is moving. In contrast, in ornstein-uhlenbeck dynamics, *σ* is the standard deviation of a gaussian that determines how much the speed changes in that time step.^11^ A naive response strategy would assume that if a target gets confused with a distractor when they cross each other, the participant will only track one of them. However, the participant can keep track of *both* of them, and then at the end of the trial, if the query distractor is neither of them, then the participant can confidently answer ‘No’.^12^ It is still too early to comment on the conceptual or non-conceptual character of these representations.^13^ Even the recent “successes” rely on unfathomably big data and energy consumption that a human child does not require in order to learn language or make sense of their visual sense data.
